# Synchronization of Coupled Different Chaotic FitzHugh-Nagumo Neurons with Unknown Parameters under Communication-Direction-Dependent Coupling

**DOI:** 10.1155/2014/367173

**Published:** 2014-06-30

**Authors:** Muhammad Iqbal, Muhammad Rehan, Abdul Khaliq, Saeed-ur- Rehman, Keum-Shik Hong

**Affiliations:** ^1^Department of Electrical and Computer Engineering, Centre for Advanced Studies in Engineering (CASE), Islamabad 44000, Pakistan; ^2^Department of Electrical Engineering, Pakistan Institute of Engineering and Applied Sciences (PIEAS), Islamabad 44000, Pakistan; ^3^Department of Cogno-Mechatronics Engineering and School of Mechanical Engineering, Pusan National University, 2 Busandaehak-ro, Geumjeong-gu, Busan 609-735, Republic of Korea

## Abstract

This paper investigates the chaotic behavior and synchronization of two different coupled chaotic FitzHugh-Nagumo (FHN) neurons with unknown parameters under external electrical stimulation (EES). The coupled FHN neurons of different parameters admit unidirectional and bidirectional gap junctions in the medium between them. Dynamical properties, such as the increase in synchronization error as a consequence of the deviation of neuronal parameters for unlike neurons, the effect of difference in coupling strengths caused by the unidirectional gap junctions, and the impact of large time-delay due to separation of neurons, are studied in exploring the behavior of the coupled system. A novel integral-based nonlinear adaptive control scheme, to cope with the infeasibility of the recovery variable, for synchronization of two coupled delayed chaotic FHN neurons of different and unknown parameters under uncertain EES is derived. Further, to guarantee robust synchronization of different neurons against disturbances, the proposed control methodology is modified to achieve the uniformly ultimately bounded synchronization. The parametric estimation errors can be reduced by selecting suitable control parameters. The effectiveness of the proposed control scheme is illustrated via numerical simulations.

## 1. Introduction

In recent decades, behavior investigation of chaotic neurons including synchronization, particularly under external electrical stimulation (EES; e.g., deep brain stimulation), has become an important area of research in the study of clinical treatment mechanisms for neurodegenerative disorders [1, 2]. The many published reports of fascinating outcomes have, since the beginnings, attracted and inspired many additional researchers to find effectual ways of improving external therapies for patients suffering from cognitive diseases [3–6]. The famous FitzHugh-Nagumo (FHN) neuronal model has been given extensive consideration for its utility in symbolizing the dynamical behavior of neurons and complex neuronal networks under EES [7].

The subject of FHN-neuronal synchronization as a potential application in cognitive engineering has been intensively examined in the literature [8–20]. Integration of the gap junction strength in FHN neurons renders the synchronization dilemma nontrivial. And the synchronization problem becomes still more complex, once the delay terms owing to distant communication are entertained in the coupled models [21–24]. To synchronize various chaotic FHN systems, researchers have utilized different control strategies including backstepping [25], active control [26], nonlinear control [8, 9, 27], and adaptive control [20, 28, 29]. Synchronization of two identical FHN neurons of known or unknown parameters, by means of nonlinear adaptive control schemes based on fuzzy logic, neural networks, uncertainty estimator, and feedback linearization, has been investigated [8, 9, 12, 13, 20]. Recently, robust adaptive control schemes for synchronization of two or three FHN neurons of unknown model parameters have been developed as well. Synchronization of two identical neurons of unknown parameters, under uncertain stimulation currents caused by medium losses and phase shifts, has been explored by application of an adaptive control scheme [14]. Another recent work has combined the ideas of parametric adaptation and *L*_2_ gain reduction for synchronization of multiple but slightly different neurons with respect to multiple communication pathways and unknown parameters and disturbances [15]. A simple methodology for synchronization of two different coupled neurons of known parameters by application of a reference-signal-based control approach has been evaluated as well [20].

The conventional techniques for synchronization of FHN neurons are based on either designed control laws for identical neurons of known or unknown parameters or developed control strategies for different neurons of known parameters. However, two coupled neurons cannot be completely identical, and the model parameters cannot be totally known, due to biological restrictions. Furthermore, whereas the traditional techniques assume bidirectional gap junctions for the interneuronal medium, they can in fact be unidirectional, resulting in different coupling strengths for each neuron [30–32]. The effects of unidirectional gap junctions in the presence of time-delay (due to neuronal separation) cannot be ignored. All in all, the development of control strategies for synchronization of two coupled delayed chaotic neurons of different and unknown parameters, particularly under disturbances, is very challenging.

This paper analyzes the behavior and synchronization of two different coupled distant FHN neurons under unidirectional gap junctions. The strengths of the gap junctions are assumed to be different for each neuron, owing to the presence of both unidirectional and bidirectional gap junctions in the interneuronal medium. Various dynamical aspects of coupled FHN neurons, such as the effects of parametric differences, time-delays, and unidirectional gap junctions on neuronal synchronization, are investigated. The design of robust adaptive control laws for synchronization of coupled chaotic distant FHN neurons under unidirectional gap junctions is also addressed. The resultant control approach represents a novel means for synchronizing different FHN neurons of unknown parameters subject to uncertain stimulation. By utilizing integral-based control and adaptation laws to deal with the unavailable neuronal state (i.e., the recovery variable), a new adaptive control scheme is developed for synchronization of different coupled chaotic FHN neurons of unknown parameters. Motivated by experimental results [33], the proposed control scheme, unlike the traditional synchronization approaches, ensures partial synchronization of neurons in terms of their activation potentials (or membrane voltages). By utilizing the ideas of the standard Lyapunov theorem [13, 34], the proposed adaptive control scheme is modified to ensure uniformly ultimately bounded synchronization and parametric estimation errors for robust synchronization of neurons against disturbances. The results of the proposed robust adaptive control scheme for chaos synchronization of FHN neurons of different and unknown parameters are verified through numerical simulation. The main contributions of this paper can be summarized as follows.

- A model of coupled FHN neurons under both unidirectional and bidirectional gap junctions is investigated.
- The complex behavior of two different coupled neurons in a medium containing gap junctions is studied through bifurcation analysis and Lyapunov-exponential investigation.
- The idea that, by increasing the time-delay or the difference between the gap junction strengths for two neurons, the synchronization error can increase, which, further, can lead to nonsynchronous neuronal behavior, is explored.
- Based on the experimental results, a biologically understandable synchronization tool ensuring convergence of the activation potential error to zero is offered, in contrast to the conventional approaches that consider unnecessary synchronization of the recovery variable [9–20].
- The proposed synchronization control methodology fills the research gap on robust adaptive synchronization of FHN neurons of different and unknown parameters subject to disturbances.

The rest of this paper is organized as follows.  Section 2 presents the model of two coupled chaotic FHN neurons for different and unknown parameters.  Section 3 analyzes the behavior of the coupled FHN neurons.  Section 4 presents the design of nonlinear adaptive and robust adaptive control schemes for synchronization of coupled chaotic FHN neurons of different and unknown parameters.  Section 5 provides the relevant numerical simulation results.  Section 6 draws conclusions. Standard notation is used throughout the paper. The notation ||·|| symbolizes the Euclidian norm of a vector.

## 2. Model Description

Consider two coupled chaotic delayed FHN neurons (see also [7, 14]) of different and unknown parameters under uncertain EES, given by

(1) dx1dt=x1(x1−1)(1−r1x1)−y1−g1(x1−x2(t−τ))+(a1ω)cos⁡(ωt+ϕ1)+ζ1,dy1dt=b1x1,dx2dt=x2(x2−1)(1−r2x2)−y2−g2(x2−x1(t−τ))+(a2ω)cos⁡(ωt+ϕ2)+ζ2,dy2dt=b2x2,

where *x*_1_ and *y*_1_ are the states of the master FHN neuron in terms of the activation potential and the recovery variable, respectively, and *x*_2_ and *y*_2_ are the corresponding states of the slave FHN neuron. The FHN model parameters (*r*_1_, *r*_2_) and (*b*_1_, *b*_2_) are linked with the neurons' nonlinear part and recovery variable, respectively. The parameters *a*_1_ and *a*_2_ denote the amplitude of the external stimulation current for the master and the slave neurons, respectively, while *ϕ*_1_ and *ϕ*_2_ represent their phase shifts. Time and the angular frequency of the stimulation current are indicated by *t* and *ω* = 2*πf*, respectively, where *f* denotes frequency. The strength of the gap junctions for communication from the master neuron to the slave neuron is represented by *g*_1_. Correspondingly, *g*_2_ represents the strength of the gap junctions for transmission of an electrochemical signal from the slave neuron to the master neuron. The time-delay between the master and slave neurons is represented by *τ*. Disturbances at the master and slave neurons are denoted by *ζ*_1_ and *ζ*_2_.

In the present work, all of the physical quantities of FHN models (1) are assumed to be dimensionless. In modeling most of biological processes, we often know only the nominal parametric values, not the true ones, owing to the biological restrictions. The amplitudes and phases associated with the stimulation current are taken to be different due to the medium losses and different path lengths occurring during the current flow from an electrode to both of the coupled neurons. The strengths of the gap junctions differ owing to the fact that some of the communication channels are unidirectional while others are bidirectional.

**Remark 1 .**

It should be noted that all of the FHN model parameters associated with the master and slave neurons in (1) are fairly different and unknown owing to the physical limitations and the biological restrictions. Furthermore, usually, the majority of gap junctions allow bidirectional communication between two neurons. However, some permit only unidirectional transmission of a signal [30–32], responsible for different strengths of gap junctions. To capture this property, the strengths of gap junctions are taken as *g*_1_ and *g*_2_. This direction-dependent selection of gap junction strength, in contrast to the schemes available in the literature [9–24], enables a more realistic model and, as such, is a superior synchronization-study tool for coupled FHN neurons.

In the next section, we examine the behavior of coupled FHN neurons (1) in exploring the effects of neuronal-parameter difference, gap junction strength variation, and time-delay deviation on synchronization.

## 3. Behavior of Coupled FHN Neurons

**Figure 1:**
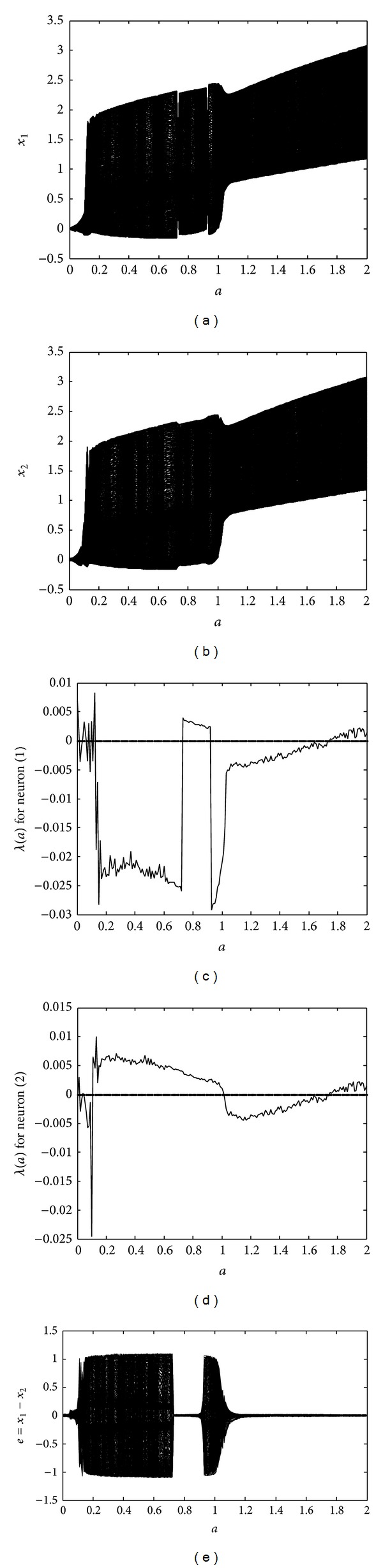
Behavior of identical FHN neurons under EES: (a) bifurcation diagram of the first neuron; (b) bifurcation diagram of the second neuron; (c) largest Lyapunov exponent for the first neuron; (d) largest Lyapunov exponent for the second neuron; (e) bifurcation diagram of the synchronization error between the coupled neurons.

Whereas, traditionally, studies have detailed the dynamical behavior of single FHN neurons, focusing on that, the dynamics of a coupled system of neurons is more significant to understanding the neuronal synchronization. Bifurcation analysis and studies on the largest Lyapunov exponent have been productive for biomedical systems such as magnetic resonance imaging of myocardial perfusion and snore classification [35, 36]. The bifurcation diagrams show the qualitative change in the dynamical behavior of neurons by changing amplitude of stimulation current over a range of 0 < *a* < 2, while the maximum Lyapunov exponent informs about how much and for which range of stimulation amplitude the neuronal behaviors are chaotic. The method of Lyapunov exponent analysis is specifically used to avoid the ambiguity that the complicated behavior is occurring either due to a strange or a chaotic attractor. Furthermore, the degree of synchronization of neurons can be quantified by utilizing bifurcation diagrams. To this end, we first select the model parameters *r*_1_ = *r*_2_ = 10.5, *b*_1_ = *b*_2_ = 1.06, *ϕ*_1_ = *ϕ*_2_ = *π*/3, *g*_1_ = *g*_2_ = 0.2, *f* = 0.135, and *τ* = 40 under disturbances *ζ*_1_ = 0.1sin12*t* and *ζ*_2_ = 0.1sin20*t* and identical stimulation amplitudes *a*_1_ = *a*_2_ = *a*, in order to study the behavior of two identical FHN neurons.  Figure 1 shows bifurcation diagrams and largest Lyapunov exponent plots for both neurons under stimulation amplitude *a*. Figures 1(a) and 1(b) indicate that both neurons exhibit oscillatory behavior for almost all values of the stimulation amplitude. Figures 1(c) and 1(d) show that the neurons exhibit chaotic behavior when the largest Lyapunov exponent becomes greater than 0. Specifically, the first FHN neuron shows chaotic behavior in the regions 0.04 < *a* < 0.13 and 0.74 < *a* < 0.92, whereas the second FHN neuron shows chaotic behavior in the region 0.12 < *a* < 1.  Figure 1(e) depicts a more interesting phenomenon, that is, synchronization of FHN neurons by means of a rare bifurcation diagram treatment of synchronization error *e* = *x*_1_ − *x*_2_. It is evident that the identical neurons possess synchronous behavior, except in the regions 0.05 < *a* < 0.74 and 0.9 < *a* < 1.1. This means that both of the neurons can be synchronized by selecting a proper stimulation amplitude, either in the region 0.74 < *a* < 0.9 or *a* > 1.1, without utilizing any control signal.

**Figure 2:**
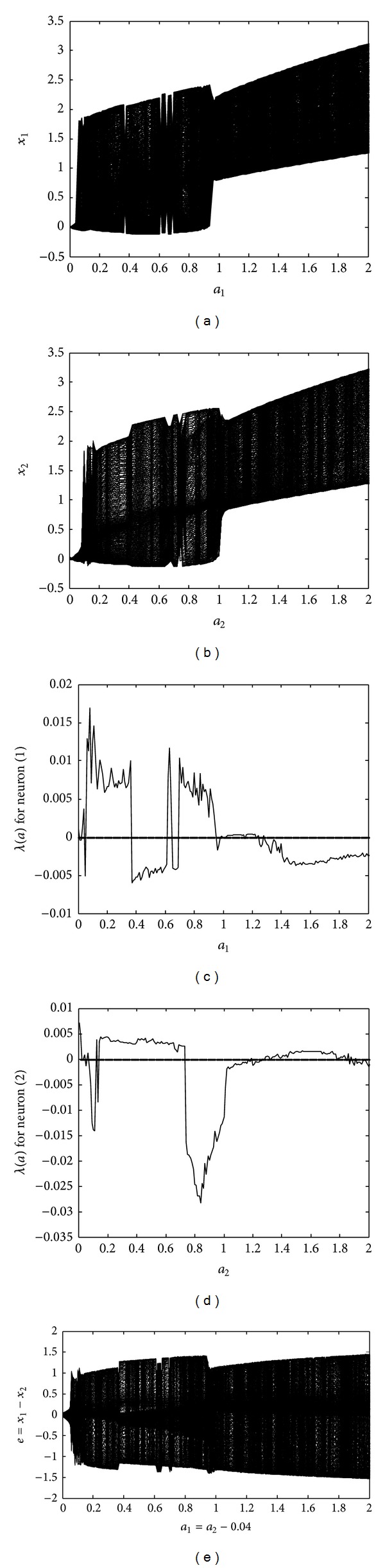
Behavior of different FHN neurons under EES: (a) bifurcation diagram of the first neuron; (b) bifurcation diagram of the second neuron; (c) largest Lyapunov exponent for the first neuron; (d) largest Lyapunov exponent for the second neuron; (e) bifurcation diagram of the synchronization error between the coupled neurons.

**Figure 3:**
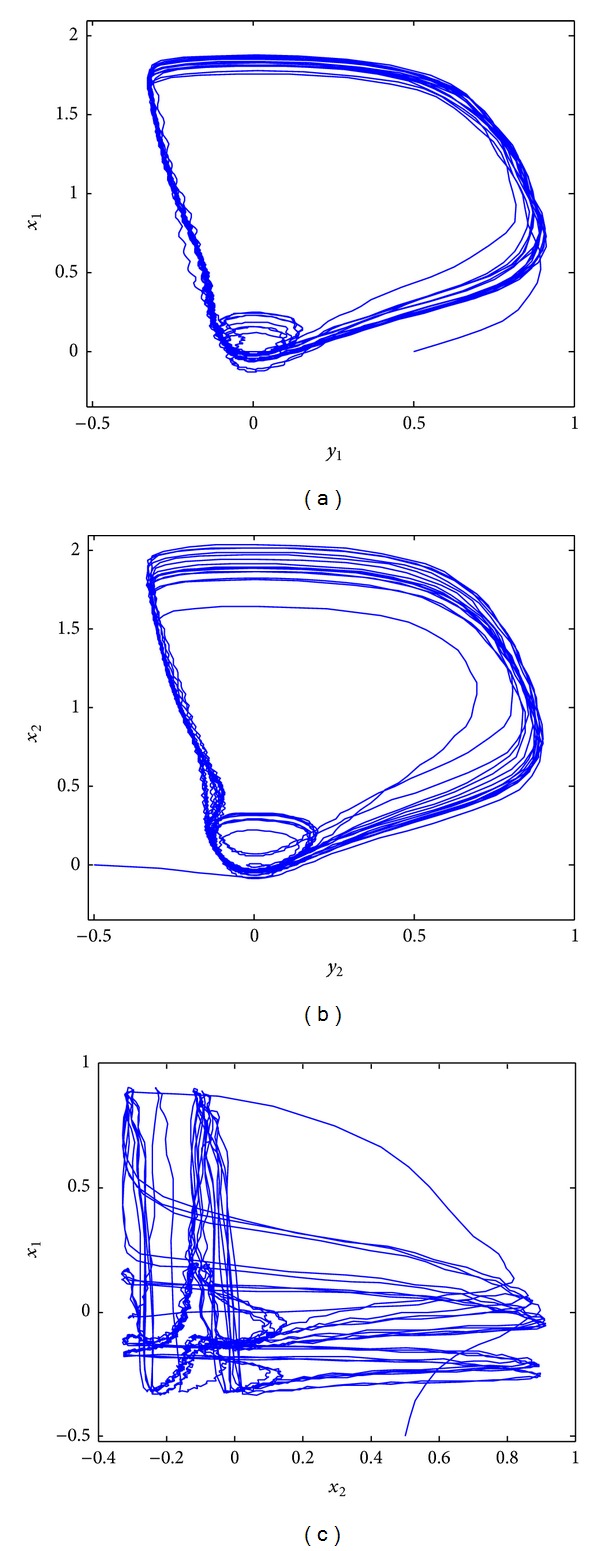
Nonsynchronous behavior of the two different FHN neurons under EES: (a) phase portrait of the first neuron; (b) phase portrait of the second neuron; (c) phase portrait of the activation potentials for nonsynchronous behavior.

Next, the dynamics of different coupled FHN neurons are analyzed by changing the parameters of the first neuron to *r*_1_ = 10, *b*_1_ = 1, *ϕ*_1_ = *π*, and *g*_1_ = 0.1. The amplitudes of stimulation are taken to be different, and the difference is fixed to *a*_2_ − *a*_1_ = 0.04.  Figure 2 plots the bifurcation diagrams and largest Lyapunov exponents for the system of different coupled FHN neurons. Similarly, to the identical neurons case, both neurons exhibit oscillatory behavior for most of the amplitude values, as shown in Figures 2(a) and 2(b). The first neuron shows chaotic behavior in the regions 0.03 < *a*_1_ < 0.37 and 0.69 < *a*_1_ < 0.95, as depicted in Figure 2(c), while the second neuron shows chaotic behavior in the regions 0.12 < *a*_2_ < 0.72 and 1.33 < *a*_2_ < 1.77, as indicated in Figure 2(d). The bifurcation diagram of synchronization error *e* = *x*_1_ − *x*_2_ is shown in Figure 2(e). Surprisingly, neither of the neurons are at all synchronous for any stimulation amplitude within the entire region 0 < *a*_1_ < 2 (and 0.4 < *a*_2_ < 2.4), due to the different parameters as compared with the case of identical neurons. We can conclude that the two different FHN neurons can be nonsynchronous, owing to variations in model parameters and, additionally, that a suitable value of stimulation amplitude in the set *a*_1_ ∈ [0 2] (correspondingly *a*_2_ ∈ [0.4 2.4]), for synchronization of these different neurons, might not exist. For further elaboration, phase portraits of the two different neurons, under the initial conditions *x*_1_(0) = 0.5, *x*_2_(0) = −0.5, *y*_1_(0) = 0, and *y*_2_(0) = 0, are shown in Figure 3 for *a*_1_ = 0.1 and *a*_2_ = 0.14. It is evident that both neurons, possessing the chaotic behavior shown in Figures 3(a) and 3(b), are not synchronous, as indicated in Figure 3(c).

**Figure 4:**
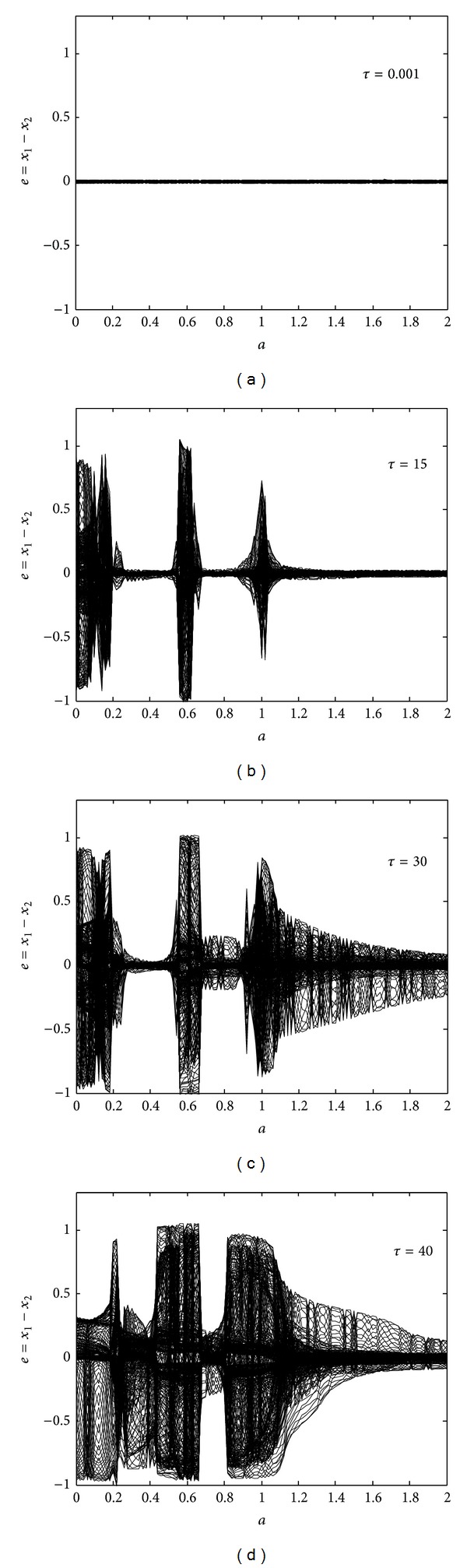
Effects of time-delay due to separation between the two neurons under different gap junction strengths: (a) bifurcation diagram of the synchronization error for *τ* = 0.001, (b) bifurcation diagram of the synchronization error for *τ* = 15; (c) bifurcation diagram of the synchronization error for *τ* = 30; (d) bifurcation diagram of the synchronization error for *τ* = 40.

**Figure 5:**
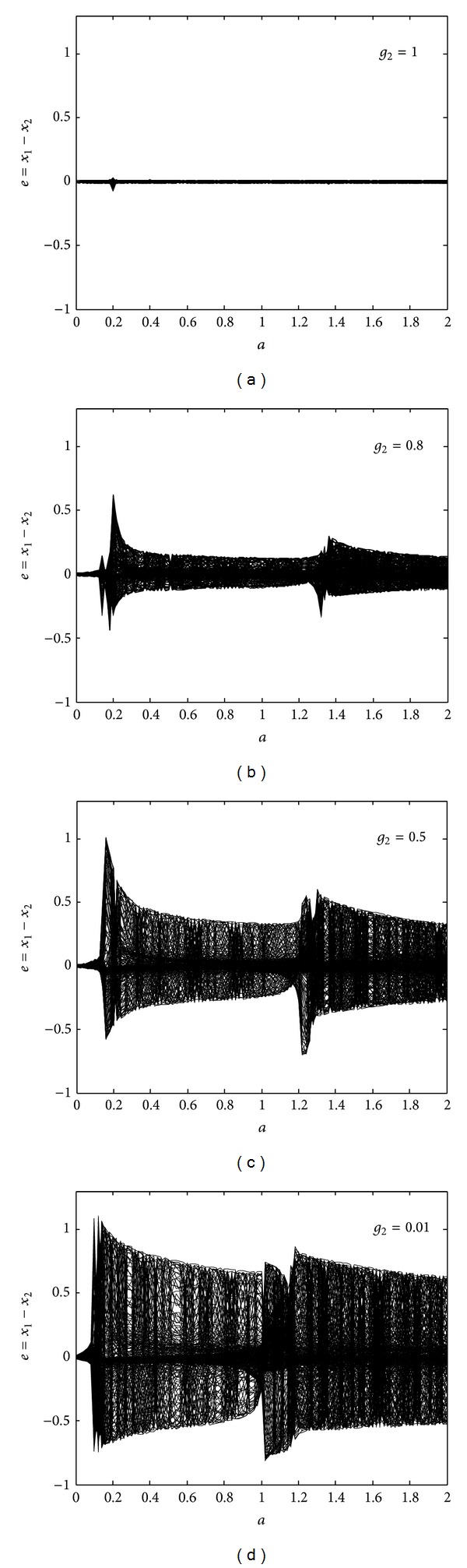
Effects of the unidirectional gap junctions in a medium between the two neurons: (a) bifurcation diagram of the synchronization error for *g*_2_ = 1; (b) bifurcation diagram of the synchronization error for *g*_2_ = 0.8; (c) bifurcation diagram of the synchronization error for *g*_2_ = 0.5; (d) bifurcation diagram of the synchronization error for *g*_2_ = 0.01.

We now examine the effects of the strengths of gap junctions and of time-delays between two identical neurons. The model parameters are selected as *r*_1_ = *r*_2_ = 10.5, *b*_1_ = *b*_2_ = 1.06, *ϕ*_1_ = *ϕ*_2_ = *π*/3, *f* = 0.135, *ζ*_1_ = 0.1sin12*t*, and *ζ*_2_ = 0.1sin20*t*, with identical stimulation amplitudes; that is, *a*_1_ = *a*_2_ = *a*.  Figure 4 shows bifurcation diagrams of the synchronization error for different values of time-delay *τ* under fixed (but different) values of gap junction strengths *g*_1_ = 0.8 and *g*_2_ = 0.9. For the small value of *τ* = 0.001, the FHN neurons are synchronous, as Figure 4(a) illustrates. As we increase the time-delay to *τ* = 15, the synchronization error, as shown in Figure 4(b), increases. The neurons exhibit nonsynchronous behavior for *τ* = 15 in the three regions 0 < *a* < 0.2, 0.5 < *a* < 0.7, and 0.95 < *a* < 1.05. The overall region of nonsynchronous behavior further increases for time-delay *τ* = 30 and becomes the largest for *τ* = 40, as shown in Figures 4(c) and 4(d), respectively.  Figure 5 provides bifurcation diagrams of the synchronization error under different values of *g*_2_ for the constant parameters *g*_1_ = 1 and *τ* = 1. At *g*_2_ = 1, the neurons behave like identical oscillators with a small synchronization error, as depicted in Figure 5(a). As we decrease the value of *g*_2_ to 0.8 and, further, to 0.5, the synchronization error increases, as shown in Figures 5(b) and 5(c), respectively. At *g*_2_ = 0.01, the FHN neurons' degree of nonsynchronization is the worst, as apparent in Figure 5(d). Important conclusions can be drawn from Figures 4 and 5: either synchronization error *e* between the activation potentials of two FHN neurons or the region of nonsynchronous behavior can increase, either for distant neurons with more time-delay *τ* or for neurons with large (absolute) difference values between gap junction strengths *g*_1_ and *g*_2_; accordingly, the behavior of two FHN neurons subject to a medium containing both unidirectional and bidirectional gap junctions can change from synchronous to nonsynchronous on an increase of either *τ* or |*g*_1_ − *g*_2_|.

## 4. Synchronization of FHN Neurons

The present work proposes a control strategy that uses a single control input *u* for synchronization of coupled FHN neurons of different and unknown parameters. Thus, model (1) takes the form

(2) dx1dt=x1(x1−1)(1−r1x1)−y1−g1(x1−x2(t−τ))+(a1ω)cos⁡(ωt+ϕ1)+ζ1,dy1dt=b1x1,dx2dt=x2(x2−1)(1−r2x2)−y2−g2(x2−x1(t−τ))+(a2ω)cos⁡(ωt+ϕ2)+ζ2+u,dy2dt=b2x2.

**Assumption 2 .**

The parameters (*r*_1_, *r*_2_, *b*_1_, *b*_2_, *g*_1_, *g*_2_, *a*_1_, *a*_2_, *ϕ*_1_, and *ϕ*_2_) of FHN neurons (2) are unknown constants.

Now, we develop a new control methodology for synchronization of master-slave neurons (2) of different and unknown parameters. Traditionally, synchronization of neurons is addressed in order to minimize the differences between all of the corresponding states of the master and slave neurons. In the literature [9–24], synchronization techniques for FHN neurons ensure convergence of both synchronization errors, the difference between the activation potentials and the error between the recovery variables for the master-slave systems, either to zero or in a small compact set. Nevertheless, various experimental studies (e.g., [33]) have demonstrated identical behavior of two synchronous neurons for their activation potentials only. In reality, the recovery variable is introduced in the model for membrane responses of potassium activation and sodium inactivation. Two different FHN neurons, with identical firing in terms of membrane (or activation) potentials, might not necessarily have the same (or similar) patterns for this hypothetical recovery variable. This property can also be verified from FHN neurons (2). Suppose that control law *u* is designed to achieve *x*_1_ = *x*_2_ = *x* and (2) reveal that ${\dot{y}}_{1}=b_{1}x$ and ${\dot{y}}_{2}=b_{2}x$. This implies that the recovery variables, due to different parametric values of *b*_1_ and *b*_2_, are not identical for the two different neurons. In fact, different behavior between the recovery variables can be responsible for identical membrane potentials of two different neurons.

Here, we address a partial synchronization of two distinct FHN neurons according to their activation potentials, as supported by experimental results and theoretical reasoning. In order to construct a control law, the dynamics of synchronization error *e* = *x*_1_ − *x*_2_ for FHN neurons (2) can be expressed as

(3) dedt=f1(x1)−f2(x2)−y1+y2−g1(x1−x2(t−τ))+g2(x2−x1(t−τ))+(aCφω)cos⁡ωt−(aSφω)sinωt+ζ−u,

where

(4) f1(x1)=−r1x13+r1x12+x12−x1,f2(x2)=−r2x23+r2x22+x22−x2,aCφ=a1cos⁡φ1−a2cos⁡φ2,aSφ=a1sinφ1−a2sinφ2,ζ=ζ1−ζ2.

The synchronization error dynamics in (3) contain the recovery variables (i.e., *y*_1_ and *y*_2_). These terms can be canceled through the control law *u*; however, this requires measurement (or estimation) of the recovery variables, which may not be possible in the case of neuronal synchronization. To deal with this problem, we integrate the recovery-variable dynamics in (2) to obtain

(5) y1=b1∫0tx1dα+y1(0),y2=b2∫0tx2dα+y2(0).

According to (4)-(5), the alternate synchronization error dynamics becomes

(6) $$\begin{array}{c} \frac{de}{dt}=\Phi^{T}\Gamma \left(x_{1},x_{2}\right)+x_{1}^{2}-x_{2}^{2}-e+\zeta -u, \end{array}$$

where Φ ∈ *R* ^10^ is a vector of unknown constant parameters and Γ(*x*_1_, *x*_2_) ∈ *R* ^10^ is a vector of a known bases function given by

(7) ΦT=[r1r2b1b2y1(0)y2(0)g1g2aCϕaSϕ]

(8) Γ(x1,x2)=[−x13+x12x23−x22−∫0tx1dα∫0tx2dα−11−(x1−x2(t−τ))(x2−x1(t−τ))cos⁡ωtω−sinωtω]T.

The proposed controller takes the form

(9) $$\begin{array}{c} u={\hat{\Phi }}^{T}\Gamma \left(x_{1},x_{2}\right)+x_{1}^{2}-x_{2}^{2}+Ke, \end{array}$$

where $\hat{\Phi }$ is the estimate of vector Φ and *K* is a scalar quantity. The selected adaptation law for $\hat{\Phi }$ is

(10) $$\begin{array}{c} \dot{\hat{\Phi }}=\frac{pe\Gamma \left(x_{1},x_{2}\right)1}{q},\;p>0,\;\;q>0, \end{array}$$

where *p* and *q* are scalars.

**Remark 3 .**

It is notable that the control and adaptation laws, containing integral terms in Γ(*x*_1_, *x*_2_), do not require measurement of recovery variables for the master-slave neurons, owing to the utilization of (5).

Now, we provide a condition for synchronization of FHN neurons (2) by application of control and adaptation laws (7)–(10) as follows.

**Theorem 4 .**

Consider the time-invariant FHN neural oscillators (2) with synchronization error dynamics (6)–(8) satisfying Assumption 2 under zero disturbances (i.e., *ζ*_1_ = *ζ*_2_ = 0). Nonlinear control and adaptation laws (8)–(10), which satisfy *p*(*K* + 1) > 0, ensure

- synchronization of the coupled FHN neurons under different and unknown parameters by guaranteeing the convergence of synchronization error *e* to zero;
- convergence of $\hat{\Phi }$ to ${\hat{\Phi }}^{*}$, where ${\hat{\Phi }}^{*}$ is the constant steady-state value satisfying ${\left(\Phi -{\hat{\Phi }}^{*}\right)}^{T}\Gamma (x_{1},x_{2})=0$, if the steady state is achieved within a finite time.

**Proof**

Incorporating (9) into (6) leads to

(11) $$\begin{array}{c} \frac{de}{dt}={\left(\Phi -\hat{\Phi }\right)}^{T}\Gamma \left(x_{1},x_{2}\right)-\left(K+1\right)e+\zeta . \end{array}$$

Constructing a Lyapunov function candidate as

(12) $$\begin{array}{c} V\left(e,\left(\Phi -\hat{\Phi }\right)\right)=\left(\frac{1}{2}\right)\left(pe^{2}+q{\left(\Phi -\hat{\Phi }\right)}^{T}\left(\Phi -\hat{\Phi }\right)\right), \end{array}$$

with *p* > 0, *q* > 0, the time-derivative of (12) is given by

(13) $$\begin{array}{c} \dot{V}\left(e,\left(\Phi -\hat{\Phi }\right)\right)=pe\dot{e}-q{\left(\Phi -\hat{\Phi }\right)}^{T}\dot{\hat{\Phi }}. \end{array}$$

Note that ${\left(\Phi -\hat{\Phi }\right)}^{T}\dot{\hat{\Phi }}={\dot{\hat{\Phi }}}^{T}(\Phi -\hat{\Phi })$. Incorporating (11) into (13), we obtain

(14) V˙(e,(Φ−Φ^))=pe(Φ−Φ^)TΓ(x1,x2)−p(K+1)e2 −q(Φ−Φ^)TΦ^˙+peζ.

Application of the adaptation law (10) under *ζ* = 0 yields

(15) $$\begin{array}{c} \dot{V}\left(e,\left(\Phi -\hat{\Phi }\right)\right)=-p\left(K+1\right)e^{2}. \end{array}$$

Thus, convergence of synchronization error *e* to zero is ensured, which completes proof of statement (i) in Theorem 4. In practice, it has been observed that the steady state is achieved within a finite time by application of an adaptive control law such as in Theorem 4. In the steady state, the synchronization error and the neuronal states satisfy

(16) $$\begin{array}{c} \dot{e}=0\;\;e=0,\;\;x_{1}=x_{2}=x. \end{array}$$

Using *e* = 0 in (10), $\dot{\hat{\Phi }}=0$ is obtained in the steady state. This further implies that $\hat{\Phi }={\hat{\Phi }}^{*}$ is satisfied in the steady state, where ${\hat{\Phi }}^{*}$ is a constant. Similarly to the steady-state analysis in [14], applying the steady-state conditions (i.e., $\dot{e}=0$, *e* = 0, *x*_1_ = *x*_2_, and $\hat{\Phi }={\hat{\Phi }}^{*}$) to (11), we obtain

(17) $$\begin{array}{c} {\left(\Phi -{\hat{\Phi }}^{*}\right)}^{T}\Gamma \left(x_{1},x_{2}\right)=0, \end{array}$$

which completes the proof of statement (ii) in Theorem 4.

**Remark 5 .**

In contrast to the traditional synchronization methodologies [9–20], the proposed adaptive control strategy in Theorem 4 can be used for synchronization of two different FHN neurons with all parameters unknown. This feature is achieved by incorporation of the knowledge acquired in experimental studies (e.g., [33]) on synchronization of a single state (i.e., the membrane potential) of neurons (rather than both the membrane potential and the recovery variable).

We now provide conditions for robust adaptive synchronization of different FHN neurons of unknown parameters under disturbances. First, we make the following assumption.

**Assumption 6 .**

Assume that ||*ζ*|| = ||*ζ*_1_ − *ζ*_2_|| ≤ *ζ*_*m*_ and ||Φ|| ≤ Φ_*m*_, where *ζ*_*m*_ and Φ_*m*_ are positive scalars.

**Theorem 7 .**

Consider the time-invariant FHN neurons (2) with synchronization error dynamics (6)–(8) satisfying Assumptions 2 and 6. Nonlinear control laws (8)-(9) and the adaptation law given by

(18) $$\begin{array}{c} \dot{\hat{\Phi }}=\frac{\left(pe\Gamma \left(x_{1},x_{2}\right)-k_{c}||e||\hat{\Phi }\right)}{q},\;p>0,\;\;q>0,\;\;k_{c}>0, \end{array}$$

where *k*_*c*_ is a scalar, will ensure uniformly ultimately bounded synchronization error *e* and parameter estimation error $\Phi -\hat{\Phi }$, if *p*(*K* + 1) > 0 is satisfied. Moreover, the estimation error can be kept small by selecting a large value of *k*_*c*_ for a given value of *p*, and the synchronization error can be minimized by enlarging *p*(*K* + 1) for a given value of *k*_*c*_.

**Proof**

Consider Lyapunov function (12). The time-derivative of (12) was given by (14). Using (14) and (18) implies

(19) $$\begin{array}{c} \dot{V}\left(e,{\left(\Phi -\hat{\Phi }\right)}^{T}\right)=-p\left(K+1\right)e^{2}-{\left(\Phi -\hat{\Phi }\right)}^{T}\hat{\Phi }k_{c}||e||+pe\zeta . \end{array}$$

It can easily be verified under ||Φ|| ≤ Φ_*m*_ that

(20) (Φ−Φ^)TΦ^=(Φ−Φ^)T(Φ^−Φ+Φ)≥||Φ−Φ^||2−||Φ−Φ^||||Φ||≥||Φ−Φ^||2−||Φ−Φ^||Φm.

Using (19), (20), and ||*ζ*|| ≤ *ζ*_*m*_ yields

(21) V˙(e,(Φ−Φ^)T)  ≤−p(K+1)e2−(||Φ−Φ^||2−||Φ−Φ^||Φm)   ×kc||e||+peζm,

(22) V˙(e,(Φ−Φ^)T)≤−||e||(p(K+1)||e||+kc(||Φ−Φ^||−Φm2)2−kcΦm24−pζm).

Given that *p*(*K* + 1) > 0, (22) implies that $\dot{V}(e_{1},{\left(\Phi -\hat{\Phi }\right)}^{T})<0$ if either

(23) $$\begin{array}{c} ||e||>\frac{k_{c}\Phi_{m}^{2}/4+p\zeta_{m}}{p\left(K+1\right)} \end{array}$$

or

(24) $$\begin{array}{c} ||\Phi -\hat{\Phi }||>\frac{\Phi_{m}}{2}+\sqrt{\frac{\Phi_{m}^{2}}{4}+\frac{p\zeta_{m}}{k_{c}}.} \end{array}$$

Therefore, synchronization error *e* and estimation error $\Phi -\hat{\Phi }$ are uniformly ultimately bounded. The size of compact set $||\Phi -\hat{\Phi }||\leq \Phi_{m}/2+\sqrt{\Phi_{m}^{2}/4+p\zeta_{m}/k_{c}}$ can be minimized by selecting a large value of *k*_*c*_ for a given value of *p*. Similarly, the size of ||*e*|| ≤ (*k*_*c*_Φ_*m*_^2^/4 + *pζ*_*m*_)/*p*(*K* + 1) can be kept smaller by selecting a large value of *p*(*K* + 1) for a given parameter *k*_*c*_. This completes the proof of Theorem 7.

**Remark 8 .**

By application of Theorem 7, synchronization of different chaotic FHN systems of unknown parameters under disturbances can be achieved, in contrast to the traditionalistic synchronization tools, by ensuring uniformly ultimately bounded synchronization and parametric estimation errors. Further, the effect of disturbances can be minimized by selecting suitable control parameters *K*, *k*_*c*_, and *p*.

## 5. Controlled Synchronization Simulation

**Figure 6:**
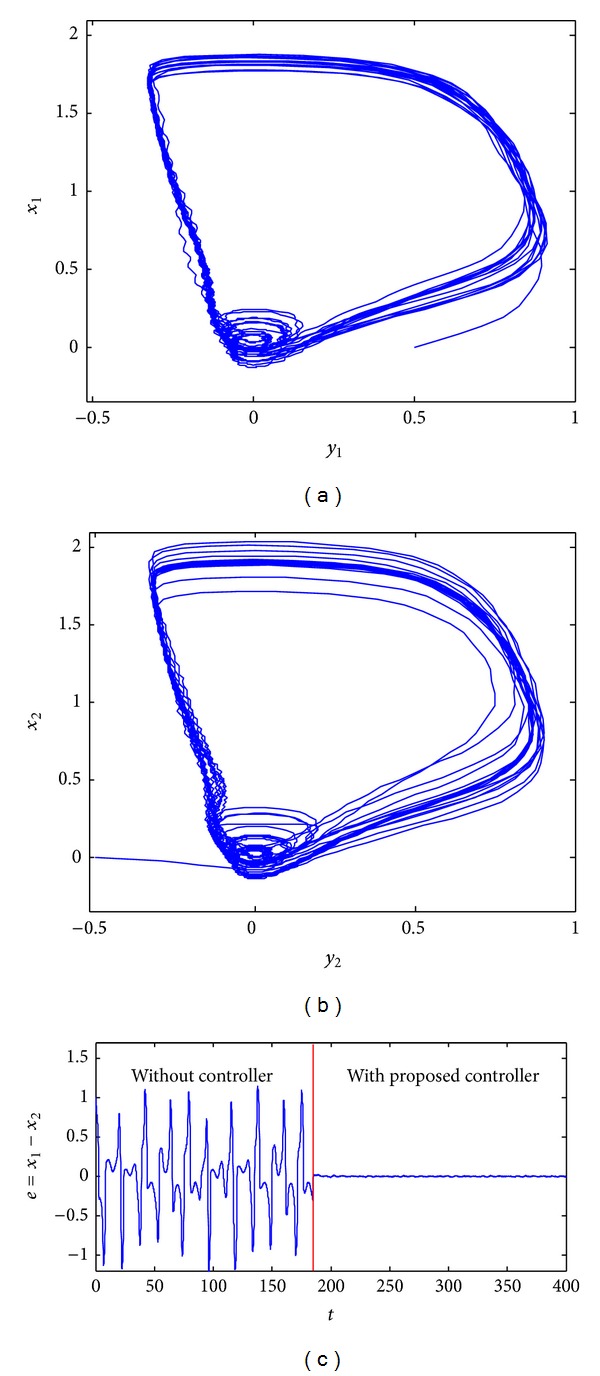
Synchronization of the two different coupled chaotic FHN neurons under EES by application of the proposed control scheme. The controller was applied for the time *t* ≥ 185: (a) phase portrait of the first neuron; (b) phase portrait of the second neuron; (c) synchronization error plot.

To demonstrate the effectiveness of the proposed methodology, we set the model parameters for FHN neurons (2) as *r*_1_ = 10,  *a*_1_ = 0.1,  *b*_1_ = 1,  *ϕ*_1_ = *π*,  *g*_1_ = 0.1,  *r*_2_ = 10.5,  *a*_2_ = 0.14,  *b*_2_ = 1.06,  *ϕ*_2_ = *π*/3,  *g*_2_ = 0.2,  *f* = 0.135, and  *τ* = 40. By Theorem 7, the controller and the adaptation law parameters are obtained as *p* = 1, *q* = 1, *k*_*c*_ = 5, and *K* = 20.  Figure 6 shows phase portraits and a synchronization error plot for the two coupled chaotic FHN neurons under disturbances *ζ*_1_ = 0.1sin12*t* and *ζ*_2_ = 0.1sin20*t*. By application of the controller at *t* = 185, synchronization error *e* converges to a small compact set, as shown in Figure 6(c).

Although the simulation results provided herein represent a specific scenario of FHN neurons, the proposed methods in Theorems 4 and 7 are applicable to a general form of FHN neurons with different parameters. Further, robustness against bounded perturbations has been ensured in Theorem 7. The results of Theorems 4 and 7 may not be applicable to FHN systems with fast time-varying parameters. Nevertheless, studies on time-varying FHN systems can be carried out in the future. To conclude, the proposed robust adaptive control methodology can be used for synchronization of distinct FHN neurons of unknown model parameters subject to disturbances.

## 6. Conclusions

This paper addressed the synchronization of two coupled chaotic FHN neurons for different and unknown parameters under uncertain external stimulation and disturbances. The dynamics of coupled FHN neurons of different parameters were studied in a medium containing both unidirectional and bidirectional gap junctions. The effects of the neuronal-parameter difference, the gap junction strength variation, and time-delay deviation on the synchronization error were investigated. Nonlinear adaptive and robust adaptive control strategies were developed to cope with synchronization of the FHN neurons under the circumstances of different and unknown parameters, the infeasibility of recovery-variable measurement, uncertainty of stimulation current, and disturbances. The proposed control scheme was successfully applied to the synchronization of coupled chaotic FHN neurons, the numerical simulation results of which were provided.
